# HBx promotes tumorigenicity through RRM2-mediated autophagy in hepatocellular carcinoma

**DOI:** 10.1186/s13578-024-01298-2

**Published:** 2024-09-10

**Authors:** Yaqun Li, Furan Wang, Zikai Geng, Tianye He, Yun Song, Jian Wu, Bin Wang

**Affiliations:** 1 Department of Pharmacy, Huashan Hospital, Fudan University, Shanghai, 200040 China; 2 Department of Pharmacy, Department of Oncology, Shanghai Medical College, Fudan University Shanghai Cancer Center, Fudan University, Shanghai, 200032 China; 3 Pfizer Research China, Shanghai, 200000 China; 4 Pharmacy School, Binzhou Medical University, Yantai, Shandong Province 264003 China; 5 National Clinical Research Center for Aging and Medicine, Huashan Hospital, Fudan University, Shanghai, 200040 China

**Keywords:** HBV, HBx, RRM2, Hepatocellular carcinoma, Autophagy

## Abstract

**Background:**

Hepatitis B virus (HBV) infection can exacerbate liver disease progression through multiple mechanisms, eventually leading to hepatocellular carcinoma (HCC). HBV-encoded oncogene X protein (HBx), a key regulatory protein of HBV infection, serves as a positive regulator of hepatocarcinogenesis. The indispensability of the M2 subunit of ribonucleotide-diphosphate reductase (RRM2) lies in its role in facilitating DNA replication and repair processes. In our previous investigation, it was postulated that the gene RRM2 exhibits elevated expression levels in several categories of malignant tumors, particularly in HBV-related HCC. Additionally, it was observed that RRM2 is present within protein complexes that are centered on HBx. In the present investigation, the objective of this work was to investigate the potential relationship between the elevated expression of RRM2 in HBV-related HCC and the influence of HBx on this expression. The study attempted to determine the specific mechanism by which RRM2 is implicated in the promotion of hepatocarcinogenesis by HBx. There have been multiple scholarly proposals suggesting that the induction of autophagy by HBx is a significant intermediary factor in the development of HCC. However, the precise carcinogenic function of HBx-induced autophagy remains a subject of debate.

**Results:**

This work initially investigated the impact of suppressing cellular autophagy on the malignant biological behaviors of HBx-promoted cells using an in vitro cellular model. The findings revealed that the suppression of cellular autophagy partially disrupted the oncogenic effects of HBx. In light of this, we proceeded to conduct more investigations into the regulatory association between RRM2 and HBx-induced autophagy in the upstream-downstream context. Our data indicate that HBx proteins increase the expression of RRM2. Suppression of RRM2 expression not only hinders HBx-induced autophagy, but also worsens the cellular G1/S blockage and reduces the HBx-induced malignant growth of hepatocellular carcinoma tumors, while stimulating apoptosis.

**Conclusions:**

Therefore, we hypothesised that RRM2 is a potential downstream target of HBx-induced hepatocarcinogenesis, and mining the oncogenic mechanism of RRM2 is significant in exploring the preventive treatment of HBV-related HCC.

**Supplementary Information:**

The online version contains supplementary material available at 10.1186/s13578-024-01298-2.

## Introduction

The phenomenon of increased expression of Ribonucleotide-diphosphate reductase M2 subunit (RRM2), a critical protein involved in the processes of DNA replication and repair, has been documented in various types of cancerous tumors [1, 2]. The atypical increase in RRM2 expression is implicated in the promotion of rapid cell proliferation through the augmentation of deoxyribonucleotide triphosphate (dNTP) accumulation [3, 4]. The dysregulation of RRM2 has been found to be correlated with elevated mortality rates in numerous cancer patients. And the prognostic significance of RRM2 has been documented in various cancer types, including pancreatic cancer and glioblastoma [5, 6]. Liu X et al. [7] observed a notable increase in the expression level of RRM2 in bone marrow mononuclear cells obtained from individuals diagnosed with multiple myeloma and there was a strong association between the expression level of RRM2 and various clinical parameters, including the International Staging System (ISS) analysis, bone destruction, and extramedullary infiltration. Yang B et al. [8] proposed that there is a positive association between elevated expression levels of RRM2 and the aggressive characteristics of endometriosis, which indicated RRM2 might potentially be utilized as a diagnostic biomarker for cervical cancer. According to Liu Q et al. [9], there is a correlation between the expression of the oncogenic transcription factor MYBL2 and the up-regulation of RRM2 expression in colon cancer, and MYBL2 is able to bind directly to the promoter region of the RRM2 gene to promote its transcription, which then regulates the proliferation mechanism of cancer cells. In addition, it has been clinically observed that a high level of RRM2 expression plays a significant role in chemotherapeutic drug resistance. Therefore, inhibition of RRM2 expression has the potential to be a feasible strategy to improve the anticancer effects of chemotherapeutic agents [10]. At present, the clinical management of glioblastoma, chronic granulocytic leukemia, acute granulocytic leukemia, and sickle cell anemia has incorporated the utilization of inhibitors that specifically target RRM2, yielding favorable therapeutic outcomes [11–13]. The compound COH29 has demonstrated significant inhibitory action against the RRM2 enzyme and has exhibited promising outcomes in phase I clinical trials as an anti-tumor agent [14, 15].

In a recent study conducted in our laboratory, a thorough examination was undertaken to assess the carcinogenic properties of RRM2 across various types of cancer. The findings of this research indicated that RRM2 has the potential to serve as a molecular biomarker for prognosticating outcomes and predicting the effectiveness of immunotherapy in pan-cancer, with a particular focus on HCC [16]. Furthermore, we conducted a comprehensive LC-MS/MS component analysis to predict protein complexes associated with HBx in hepatocellular carcinoma cells [17]. Our previous research yielded significant evidence suggesting a potential correlation between RRM2 and HBx at the protein level [17]. Nevertheless, there is a dearth of investigation into the oncogenic role and inherent oncogenic mechanism of RRM2 in HBV- related HCC, which is the primary focus and objective of present study.

It is evident that the oncogene X protein (HBx) encoded by HBV contributes to hepatocarcinogenesis, although current research is unable to elucidate the specific mechanisms by which HBV causes hepatocarcinogenesis [18, 19]. Wang Y et al. [20] proposed that HBx stimulates HBV replication by inducing HSPA8 expression, which in turn promotes the development of HCC by inhibiting ferrocyte prolapse. Chaomin W et al. [21] hypothesized that the regulatory effect of HBx on SMAD4 is necessary for the proliferation of HBV-related HCC cells. Recent research has indicated that alterations in cellular function induced by HBx are closely associated with the regulation of cellular autophagy. This association is particularly evident in the abnormal proliferation, differentiation, transformation, and apoptosis of cells resulting from the activation of different oncogenes during the development of HCC [22, 23].

Autophagy is a cellular process that maintains homeostasis by digesting dysfunctional organelles and proteins within cells. This mechanism plays a significant role in the progression of several types of cancers [24]. Previous research has suggested that autophagy functions as a double-edged weapon in the progression of HCC, and its oncogenic role in the development and progression of HCC remains controversial. In mice, suppression of BECN1 decreased autophagic activity and increased the incidence of HCC, according to Yue Z et al. [25]. However, Siddharth S. et al. proposed that inhibition of cytoprotective autophagy improves the efficacy of chlordecone A, an effective drug for inhibiting HCC, while induction of autophagy promotes the development of hepatic nodules in early preneoplastic rats [26]. Tang et al. proposed that HBx has the potential to enhance LC3-II expression in an in vitro setting, leading to an upregulation of BECN1 and subsequently promoting cellular autophagy [27]. Zhang Y et al. hypothesized that HBx-induced autophagy upregulates the level of TGF-1 in HCC, which promotes invasion and metastasis of HCC [28].

Consequently, the first objective of this study was to validate the significance of autophagy as a crucial intermediary mechanism in the progression of hepatocellular carcinoma induced by HBx. And based on these ofindings, we conducted an investigation into the potential association between RRM2 and HBx-induced autophagy, as well as the involvement of RRM2 in the progression of HBV-related HCC. Specifically, we examined the control of cell cycle, cell proliferation, and apoptosis by RRM2 in response to HBx stimulation. Our results demonstrated that HBx could increase the level of cellular autophagy by promoting the high expression of RRM2, which in turn promoted the malignant proliferation of hepatocyte hepatocytes and inhibited the apoptosis of HCC cells, leading to the development of liver carcinogenesis. The aforementioned findings indicate that RRM2 may have a significant function in encouraging cancer development in HBV-related HCC. Additionally, it has the potential to be utilized as a diagnostic tool and a target for preventative therapeutic interventions in HBV-related HCC.

## Results

### RRM2 as a potential tumor promoter in HCC

**Fig. 1:**
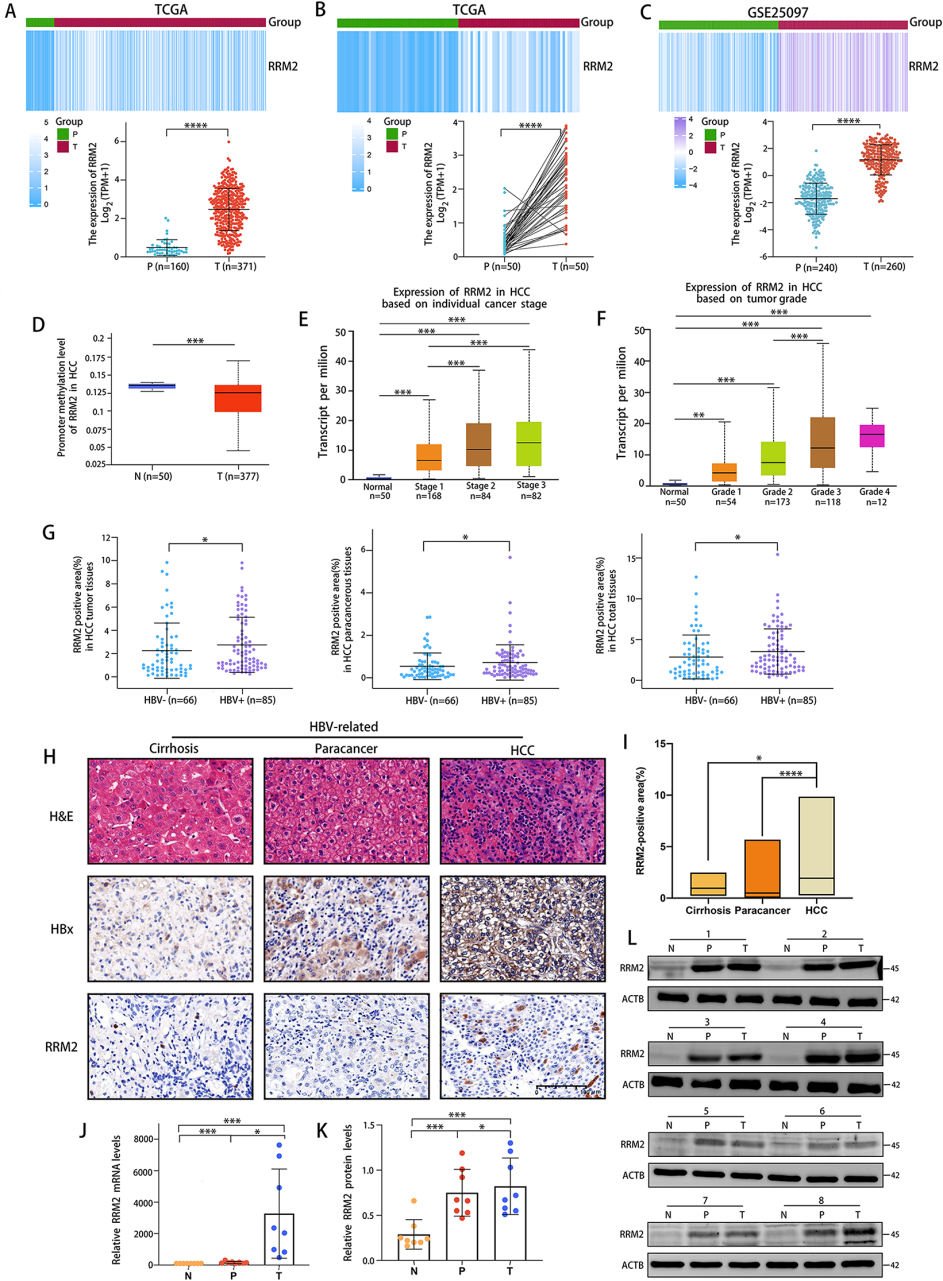
RRM2 is involved in HBV-related HCC. Boxplot **(A)** and Pairwise boxplot **(B)** of RRM2 expression between HCC tumor tissues (T) and pericarcinomatous tissues (P) using heatmaps from TCGA datasets. **(C)** The expression of RRM2 in HCC tumor tissues was compared with that in the pericarcinomatous tissues in the GSE25097 dataset. **(D)** RRM2 methylation between HCC tumor tissues and normal tissues in UALCAN dataset. **(E)** Association between RRM2 mRNA expression and specific cancer stages in HCC patients of UALCAN dataset. **(F)** Association between RRM2 mRNA expression and tumor grade in HCC patients of UALCAN dataset. **(G)** Positive area of RRM2 staining in HBV (-) or HBV (+) HCC tumor tissues and pericarcinomatous tissues, respectively and the overally. **(H)** Representative images of H&E staining, as well as HBx and RRM2 IHC staining in HBV-related liver cirrhosis tissues (*n* = 10), HBV-related HCC tissues and its corresponding pericarcinomatous tissues (*n* = 85). Scale bar: 100 μm. **(I)** Positive areas of RRM2 staining in specified tissues. **(J)** Relative RRM2 mRNA levels in human normal liver tissues (N), HBV-related HCC tissues (T) and its corresponding pericarcinomatous tissues (P) by qRT-PCR. *n* = 8 in each group. **(K**,** L)** RRM2 protein levels in indicated human liver tissues were determined by WB. ACTB was used as the loading control. *n* = 8 in each group. *P < 0.05, **P < 0.01, ***P < 0.001, ****P < 0.0001

Firstly, RRM2 expression level in HCC was analyzed using the Cancer Genome Atlas (TCGA) and Gene Expression Omnibus (GEO) datasets. The results showed that the expression of RRM2 was upregulated in HCC tissues compared with noncancerous tissues (Fig. 1A–C). However, the methylation level of RRM2 decreased in 377 TCGA-LIHC patients, in contrast to the expression level (Fig. 1D). As illustrated in Fig. 1E, RRM2 overexpression was strongly linked with advanced patient cancer stages, with the highest RRM2 expression observed in stage 3, which corresponds to the degree of vascular invasion. Similarly, RRM2 expression increased as tumor grade increased. The highest RRM2 mRNA expression was seen in grade 4 tumors (Fig. 1F). HBx and RRM2 protein expression was observed by Immunohistochemistry (IHC) staining in 10 HBV-related cirrhosis tissues, with 151 pairs of HCC tumor tissues and adjacent paraneoplastic tissues (85 of which were HBV-related HCC). We discovered that RRM2 expression was significantly higher in HBV-positive HCC tissues compared to HBV-negative HCC tissues, and this tendency was also observed in the noncancerous tissues that correspond to cancerous tissues, which implies that HBV could promote the expression of RRM2 (Fig. 1G). In HBV-related cirrhosis, HBV-related HCC, and its corresponding noncancerous tissues, obvious HBx expression was displayed, and strong positive RRM2 staining was much deeper in HCC tissues than in cirrhosis tissues (Fig. 1H, I). In addition, the expression of RRM2 also depicted an upward trend between cirrhosis and HCC in GSE10143, GSE54236, GSE25097 and GSE17548 datasets from GEO database (Supplementary Fig. S1). Moreover, the RRM2 mRNA level was higher in HBV-related HCC tissues and its corresponding adjacent noncancerous than in normal liver tissues (Fig. 1J-L). These results suggested that RRM2 acted as a possible tumor promoter in HBV-related HCC.

### High RRM2 expression was responsible for a poor prognosis in HCC patients

**Fig. 2:**
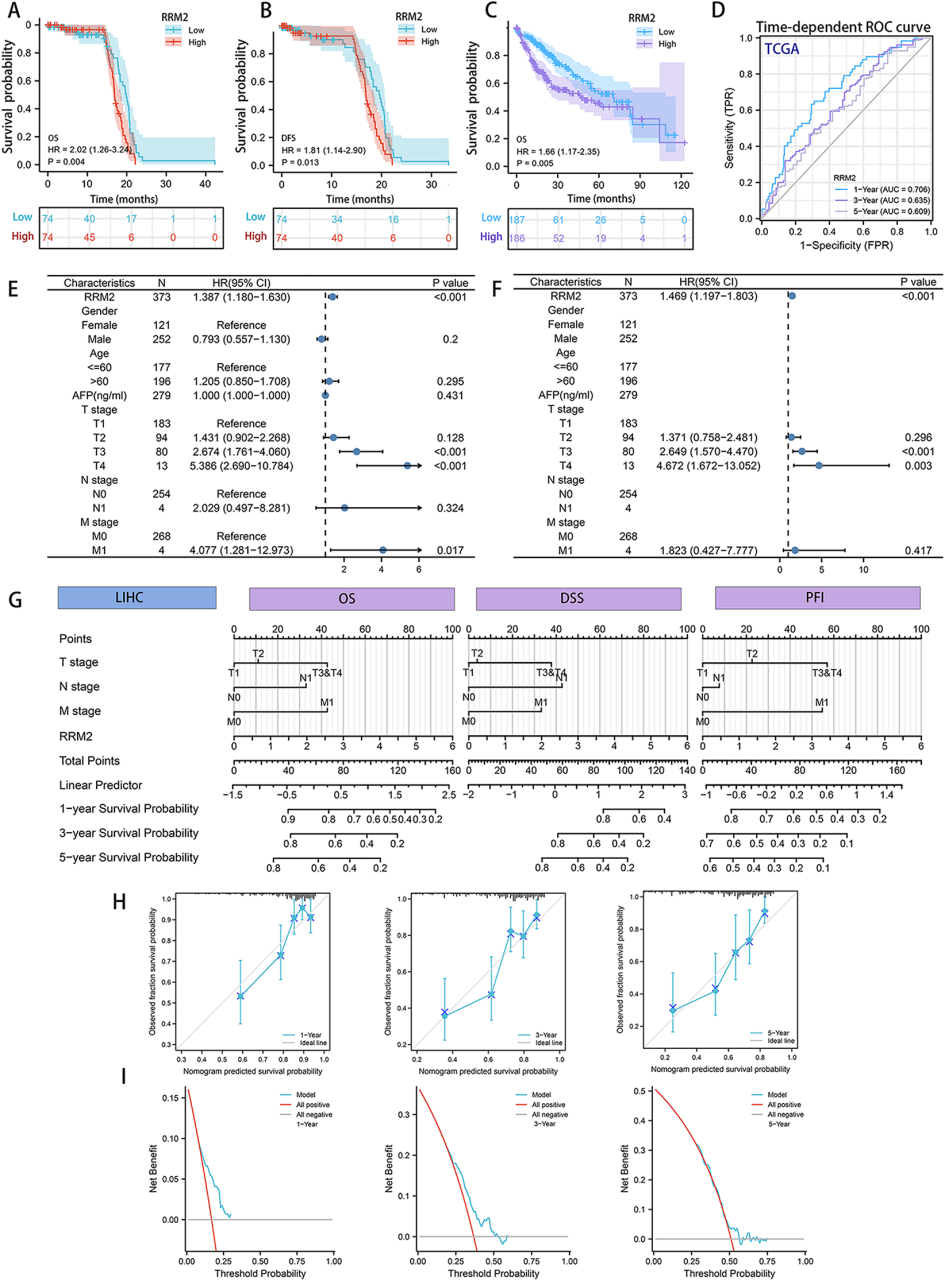
Increased RRM2 expression was related with a bad outcome in HCC patients. **(A**,** B)** Patients with high-expression levels of RRM2 had shorter OS and DFS times than low RRM2 expression patients in 148 samples. **(C)** Based on the TCGA dataset, the Kaplan-Meier survival curves depict the OS curves for HCC patients with high and low levels of RRM2 protein expression. **(D)** Time-dependent ROC curve analysis to assess the predictive efficacy of the prognostic signature. Patients with HCC from TCGA dataset were evaluated using univariate **(E)** and multivariate **(F)** Cox proportional hazards analysis to figure out the Hazard ratio (HR) of RRM2 for OS. **(G)** Nomogram for predicting the probability of 1-, 3-, and 5-year OS, DSS and PFI for HCC patients. **(H)** Calibration curve for predicting the probability of 1-, 3-, and 5-year OS for HCC patients. **(I)** DCA plots of the nomogram for predicting the probability of 1-, 3-, and 5-year OS for HCC patients by calculating the C-index

Since RRM2 was involved in HBV-related HCC, we investigated the clinical significance of RRM2 in patients with HCC. On the RRM2 positive area revealed by immunohistochemical analysis, 148 HCC patients were separated into groups with low and high RRM2 expression. Kaplan–Meier survival analysis demonstrated that higher RRM2 levels were associated with a shorter Overall survival (OS) and Disease free survival (DFS) time than lower RRM2 levels (*P* = 0.004, *P* = 0.013, respectively; Fig. 2A, B). Identical outcomes were observed in the HCC group from TCGA dataset (*P* = 0.005, Fig. 2C). In addition, we conducted a time-dependent ROC curve analysis, which revealed that RRM2 was able to distinguish tumor samples from normal ones and had a satisfactory performance in predicting survival (Fig. 2D). Figure 2E and F depicted the forest plot of univariate and multivariate Cox regression, which indicated that higher RRM2 expression was linked with poorer survival in HCC patients from TCGA dataset as well. As illustrated in Fig. 2G, we created a nomogram to estimate and manage patient efficacy based on the chance of individual survival. The C-indices of the OS, DSS, and progression-free interval (PFI) nomograms were 0.711, 0.79, and 0.633, respectively, showing that the projected results approximated the observed results to a degree. The Calibration plot displayed an approaching straight line, showing a significant correlation between the actual and projected probability at 1.3 and 5 years (Fig. 2H). DCA found that the nomogram model had a better net benefit and a higher probability threshold, indicating that it had clinical usefulness (Fig. 2I).

Then, we searched in the cBioPortal database to determine the types and frequencies of RRM2 mutations. Genes altered in 11.85% of 363 HCC patients, with mRNA upregulation occurring in 34 cases (9.37%), amplification occurring in 6 cases (1.65%), and multiple alterations occurring in 3 cases (0.83%; Fig. S2A). Thus, the observed genetic variation of RRM2 in HCC was primarily due to amplification. In comparison to the diploid group, the amplification group exhibited significantly higher levels of RRM2 expression (*P* < 0.001; Fig. S2B, C). Additionally, the Kaplan-Meier plot showed an association between RRM2 CNV and a shorter OS (log-rank test, *P* = 0.0156), a shorter DSS (log-rank test *P* = 0.0284), and a shorter progression free survival (PFS, log-rank test *P* = 6.241E-3) in HCC patients (Fig. S2D-F). These findings suggested that RRM2 and its genetic variants may also have a noticeable impact on HCC patients’ prognosis.

### Analysis of RRM2 biological significance and potential molecular mechanism in HCC

**Fig. 3:**
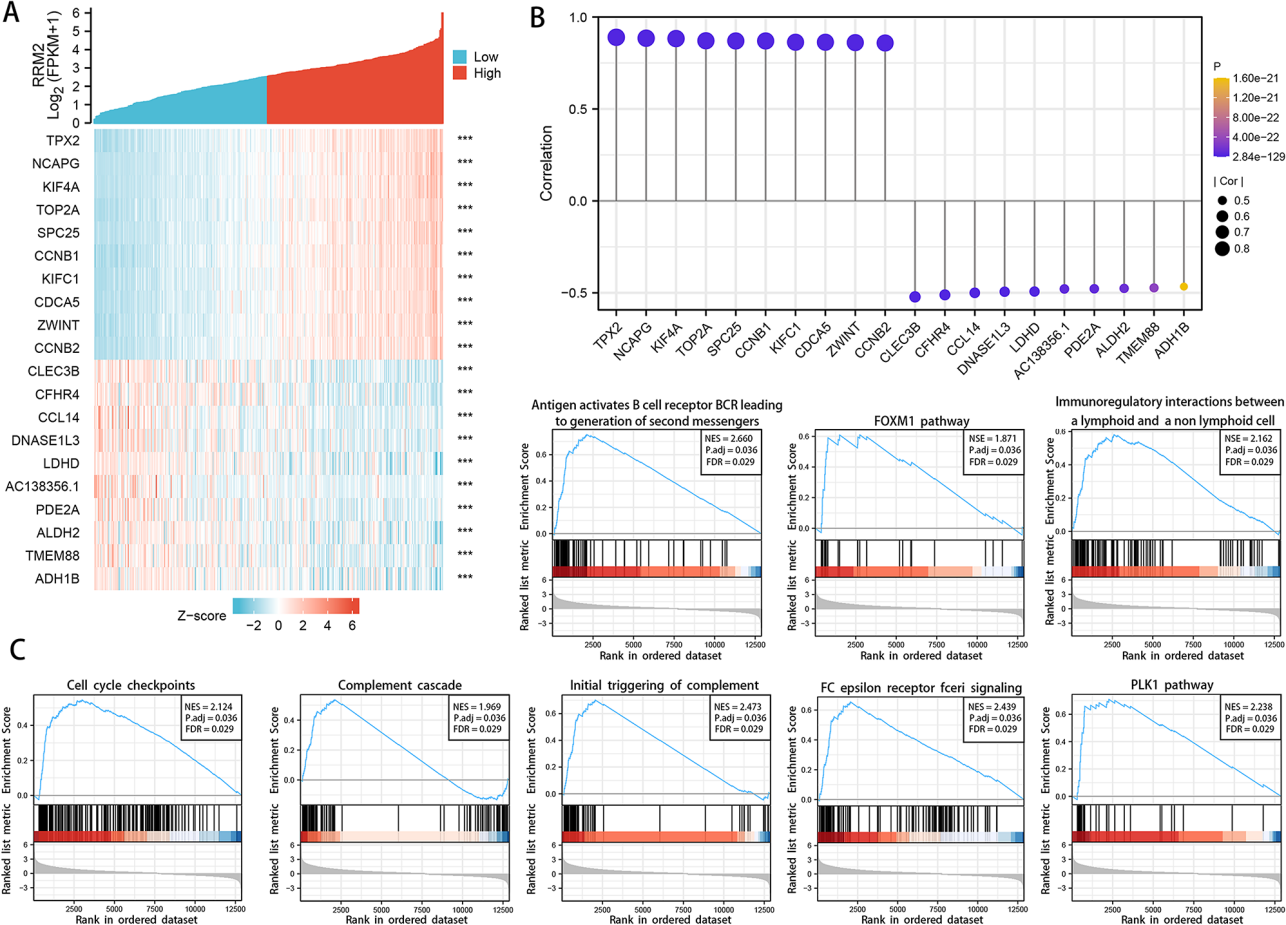
Analysis of RRM2 biological significance and potential molecular mechanism in HCC. **(A)** Heat map of top 10 DEGs in RRM2-high expression samples and low samples groups. **(B)** Expression correlation between RRM2 and representative genes (CCNB1, CDCA5, CCNB2, KIF4A, KIFC1, NCAPG, SPC25, TOP2A, TPX2, ZWINT) of the top RRM2 positive correlated genes in TCGA projects as determined. **(C)** GSEA of RRM2 in HCC. The 8 significant pathways of RRM2 GSEA results in HCC

Based on the TCGA dataset, a total of 697 DEGs were identified as statistically different between HCC RRM2-high samples and low controls. Figure 3A depicted the relative expression values of each group’s top ten DEGs. As seen in Fig. 3B, the expression of RRM2 was positively associated with that of CCNB1 (*R* = 0.877), CDCA5 (*R* = 0.884), CCNB2 (*R* = 0.873), KIF4A (*R* = 0.909), KIFC1 (*R* = 0.877), NCAPG (*R* = 0.914), SPC25 (*R* = 0.0.897), TOP2A (*R* = 0.884), TPX2 (*R* = 0.903), ZWINT (*R* = 0.879) genes (all *P* < 0.001). The result of GSEA analysis exhibited that high expression of RRM2 was clearly involved in a multitude of carcinogenic signal, such as cell cycle, PLK1 pathways, FOXM1 pathways, and P53 pathways (Fig. 3C).

### HBx promoted HCC cell tumorigenesis via autophagy

**Fig. 4:**
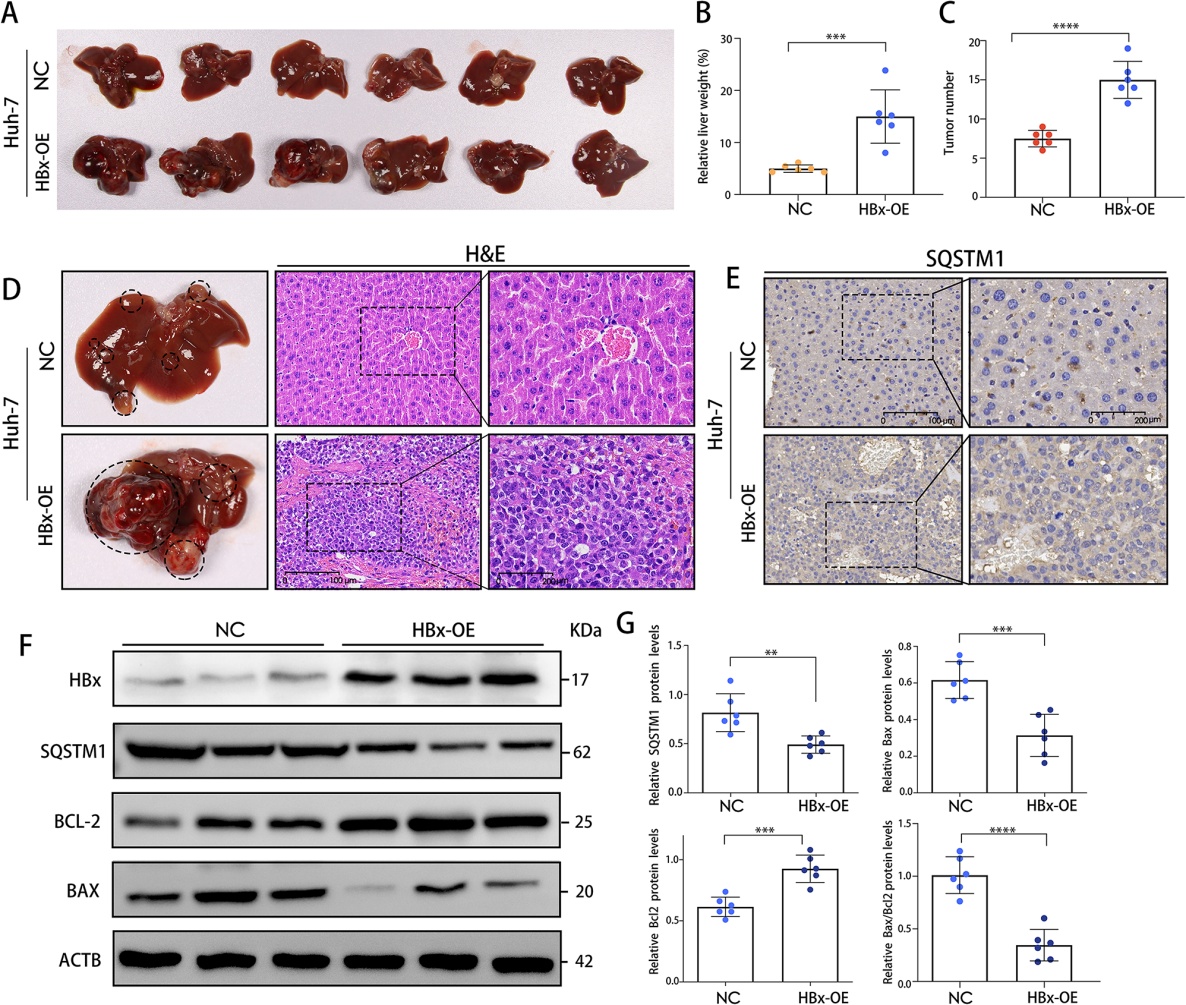
HBx promoted HCC cell tumorigenesis and autophagy. **(A)** Images of the tumors of nude mice from the NC or HBx-OE group. **(B)** The average liver weight as a percentage of total body weight. **(C)** The average tumor number of liver tissues as quantified. **(D)** Examples of liver tissues from the NC group or HBx-OE group stained with H&E. Scale bars: 100–200 μm. **(E)** Representative images of SQSTM1 staining in liver tissues from two groups. Scale bars: 100–200 μm. **(F**,** G)** HBx, SQSTM1, BCL2 and Bax protein expression in liver tissues from two groups were determined by WB. Quantification of proteins was shown in the right panel. **P < 0.01, *** *P* < 0.001, ****P < 0.0001

To confirm the oncogenic activity of HBx and its effects on HCC tumorigenicity, orthotopic xenograft tumor models were firstly developed in nude mice using Huh-7 liver cancer cells lines transfected with either LV-HBx or LV-NC. Six weeks following injection, all null mice developed orthotopic xenograft tumors at the injection site, which were collected. We found that HBx played a significant role in encouraging tumor growth, and the “primary tumors” were shown in Fig. 4A. The relative liver weights were significantly higher in the HBx-OE group than in the NC group (Fig. 4B). Moreover, the number of tumors was much lower in the NC group than in the HBx-OE group (Fig. 4C). Histopathological examination of tumor tissues indicated that they were hepatocellular carcinogenesis (Fig. 4D). WB analysis revealed that increased HBx expression was associated with decreased Bcl2 levels and increased Bax expression, indicating that apoptosis inhibition may be a potential cause of liver tissue carcinogenesis (Fig. 4F, G).

**Fig. 5:**
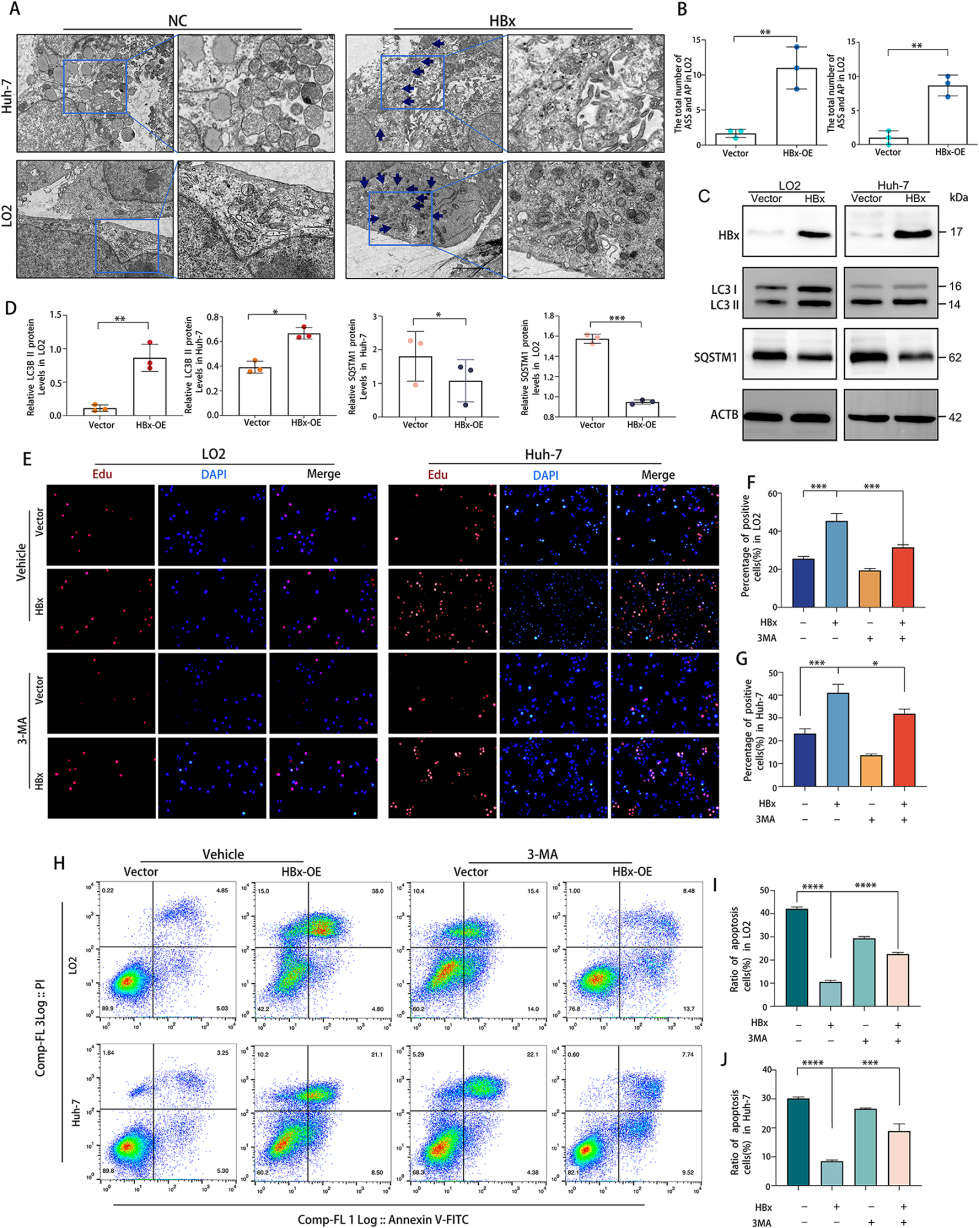
HBx promoted HCC tumorigenesis via autophagy. (**A**, **B**) Autophagosomes and autolysosomes were observed in LO2 and Huh-7 cells transfected with HA-HBx plasmid or empty NC. Scale bar, 200 nm. **(C)** The expression of the autophagy-associated proteins, LC3B, and SQSTM1 in LO2 and Huh-7 cells were detected using the corresponding antibodies with ACTB as the control by WB. **(D)** Relative expression levels of the indicated proteins in different groups (*n* = 3). LO2 or Huh-7 cells transfected with the HA-HBx construct or empty NC were treated with/without 3-MA (5 mM) for 24 h. **(E)** After treatment, the EdU incorporation assay was used to examine cell proliferation. Scale bar: 200 μm. (**F**, **G**) Quantification of positive cells in the EdU assay. (**H**-**J**) Using Annexin V-FITC/PI assay, the apoptotic index of cells subjected to diverse treatments was determined. Flow cytometry was used to examine the results (*n* = 3). *P < 0.05, **P < 0.01, ***P < 0.001, ****P < 0.0001

Autophagy is a crucial process that breaks down cellular components to maintain balance in HCC. It serves various purposes, but the exact way it responds to the tumor-inducing effects of HBx is not yet understood [29]. So, we firstly proved that HBx could promot autophagy in HCC. Following that, we investigated the role of autophagy in HBx-induced growth of tumors by examining the effects of autophagy on the proliferation and apoptosis of LO2 and Huh-7 cells transfected with LV-HBx or LV-NC. WB analysis showed the relative SQSTM1 protein expression level in the HBx-OE group was reduced compared with that of the NC group (Fig. 4F, G). Consistently, IHC analysis further showed the same results as depicted in Fig. 4E. Electron microscopy showed that the number of typical autophagosomes with double membranes was significantly increased in HBx-expressing LO2 (LO2-HBx) and HBx-expressing Huh-7 cells (Huh-7-HBx) (Fig. 5A, B). additionally, the HBx overexpression increased the conversion of LC3-I into LC3-II and decreased SQSTM1 protein, an autophagic flux marker, in LO2 and Huh-7 cells (Fig. 5C, D). Using a EdU incorporation assay, we analyzed the impact of autophagy on the proliferation of Huh-7-HBx cells and LO2-HBx cells. As demonstrated in Fig. 5E-G, HBx overexpression stimulated the proliferation of LO2 and Huh-7 cells. Whereas, treatment with the autophagy inhibitor 3-MA inhibited the HBx-induced proliferation. Multiple investigations have demonstrated that autophagy may precede apoptosis [23]. To determine whether HBx-related autophagy was associated with apoptosis in HCC cells, we measured the level of apoptosis in LO2-HBx cells and Huh-7-HBx cells using flow cytometry with Annexin V and PI staining after autophagy inhibition. As depicted in Fig. 5H-J, the HBx overexpression group experienced a significantly lower apoptosis rate than the control group. However, when HBx-overexpressed cells were treated with 3-MA to inhibit autophagy, the rate of apoptosis rose. Taken together, these findings indicated that HBx induced autophagy, which promoted the formation liver cancer.

### HBx stimulated RRM2 expression in HCC

**Fig. 6:**
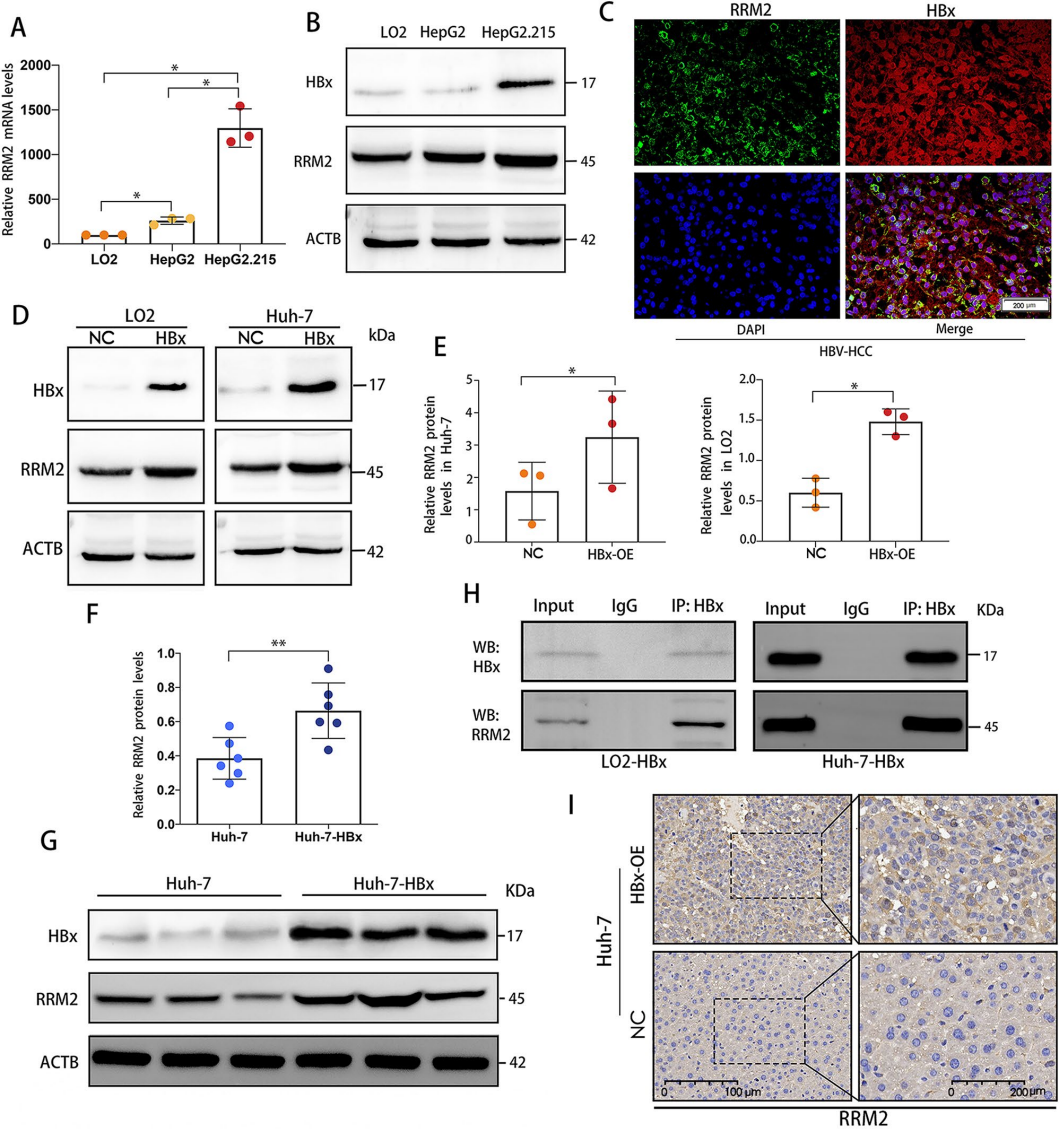
HBx stimulated RRM2 expression in HCC. **(A)** RRM2 mRNA expression levels in LO2, HepG2, and HepG2.2.15 cells (*n* = 3). (**B)** Analysis of RRM2 protein levels in the indicated cells using WB analysis (*n* = 3). **(C)** HCC tumor tissues underwent double immunofluorescence staining for HBx and RRM2. The nuclei were dyed blue with DAPI. Scale bar: 200 μm. (*n* = 6). **(D)** The protein expression levels of RRM2 in LO2 and Huh-7 cells transfected with either LV-HBx or LV-NC plasmids were detected using the corresponding antibodies with ACTB as the control by WB. **(E)** Quantification of the relative RRM2 protein expression in cells (*n* = 3). (**F**, **G**) HBx and RRM2 protein expression in liver tissues of nude mice from the NC and HBx groups were determined by WB (*n* = 6). **(H)** Anti-HBx antibody was used to perform immunoprecipitation on the cell lysates. Anti-RRM2 antibody was used to identify endogenous RRM2 that was co-immunoprecipitated. **(I)** Representative images of RRM2 staining in liver tissues from the NC and HBx groups (*n* = 6). *P < 0.05, **P < 0.01

To investigate further the potential role of HBV on RRM2, the expression of RRM2 was analyzed in LO2, HepG2, and Hep2.2.15 cells. The results showed that RRM2 was significantly elevated both at the mRNA level and the protein level in HepG2 cells compared with LO2 cells, suggesting that RRM2 is most likely an oncogene that contributes to hepatocarcinogenesis. In the meantime, we compared the expression in HepG2 of hepatocellular carcinoma cells from the same source but with varying infection status. Compared to HepG2, the mRNA level or protein expression level of RRM2 was found to be even higher in HepG2.2.215, suggesting that HBV infection promotes RRM2 expression to some degree (Fig. 6A, B). Immunofluorescent double labeling of HBx and RRM2 showed that both proteins were expressed in numerous common locations in human HCC tissues (Fig. 6C). Moreover, we discovered that RRM2 mRNA and protein expression was markedly increased following transfection of LO2 and Huh-7 cells with the HBx plasmid (Fig. 6D, E). Meanwhile, the expression of RRM2 in the liver tissues of HBx-OE mice group were elevated to a greater extent than that of control mice group (Fig. 6G, F, I). Then, the interaction between HBx and RRM2 was analyzed in LO2-HBx cells and Huh-7-HBx cells by co-immunoprecipitation, and the results revealed that RRM2 was coupled with HBx (Fig. 6H).

### Blockade of RRM2 strongly suppressed HBx-induced autophagosome formation

**Fig. 7:**
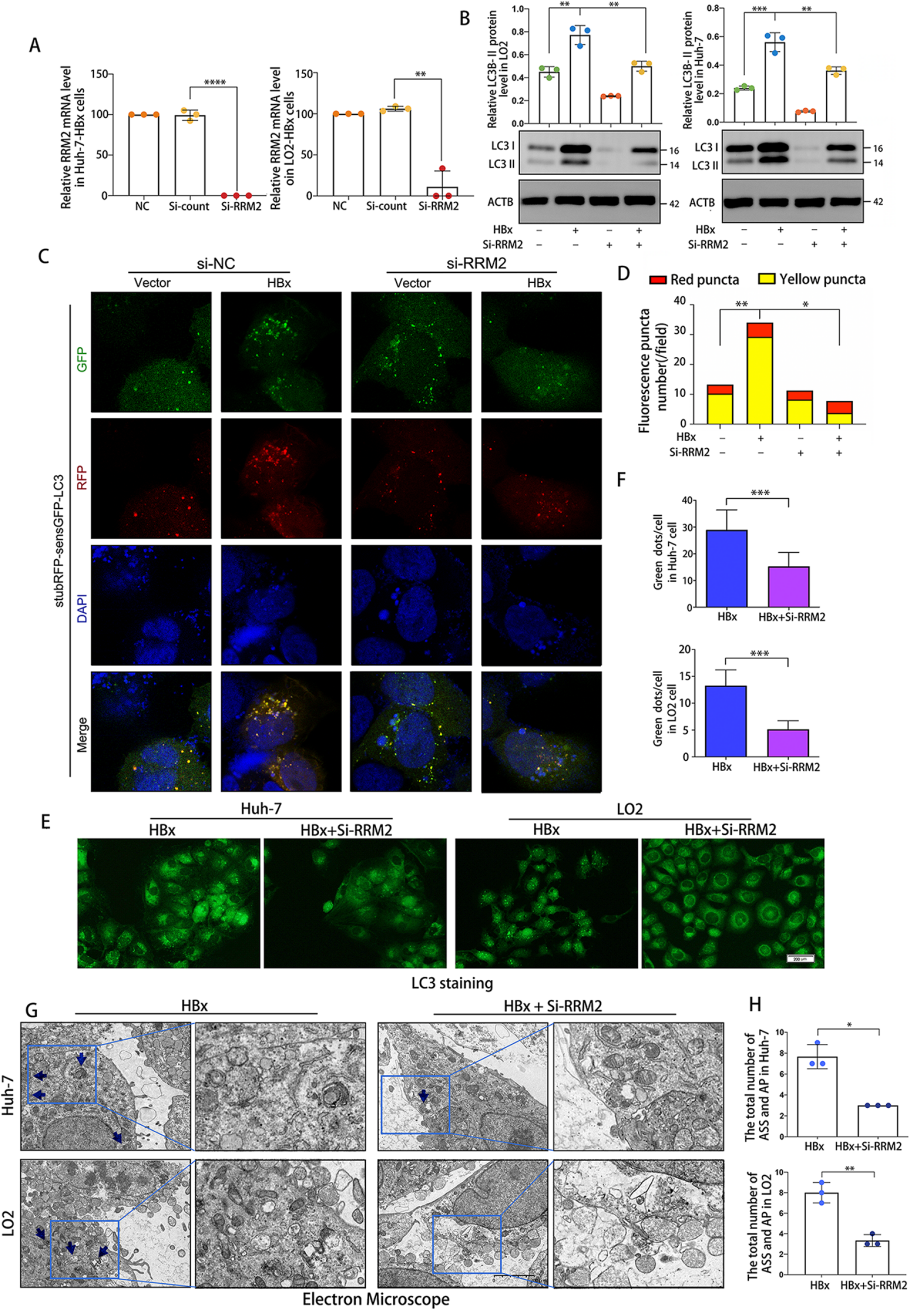
HBx-induced autophagosome production was inhibited by RRM2 blockade. LO2 or Huh-7 cells transfected with either LV-HBx or LV-NC plasmids were treated with/without siRRM2 for 48 h. **(A)** After treatment, RNA samples were then collected and relative gene expression ratios of RRM2 in cells were determined using RT-PCR analysis (*n* = 3). **(B)** Cell lysates were collected and analyzed by immunoblotting using anti-LC3B antibody. Quantification of the relative LC3B protein expression in cells (*n* = 3). **(C)** Huh-7 cells that had been genetically modified to express HBx were exposed to stubRFP-sensGFP-LC3 lentivirus, and then treated with si-RRM2 or si-NC for a period of 48 h. The nuclei are labeled with DAPI staining. Scale bar: 20 μm. **(D)** Measurement of the number of LC3 puncta in each field. **(E)** LC3 immunofluorescent staining in cells. Scale bar: 200 μm. **(F)** The mean number of LC3 puncta per cell was computed (*n* = 50 cells). **(G**, **H)** With transmission electron microscopy, double membrane autophagosome structures were identified in cells, as indicated by the arrows. Scale bar: 200 nm. *P < 0.05, **P < 0.01, ***P < 0.001, ****P < 0.0001

Our preceding data demonstrated that HBx promotes the expression of RRM2 and induces autophagy in HCC. However, the link between autophagy and RRM2 remains obscure. To further investigate the influence of RRM2 on the HBx-induced autophagic response, siRNA targeting RRM2 (si-RRM2) was transfected into LO2-HBx cells and Huh-7-HBx cells. The efficacy of RRM2 knockdown was detected 48 h after transfection (Fig. 7A). The transient transfection of si-RRM2 inhibited the HBx-induced increase in LC3B expression (Fig. 7B). In order to further investigate the impact of RRM2 on the autophagic response triggered by HBx, we utilized a stubRFP-sensGFP-LC3 reporter system for observing alterations in autophagic flux. Autophagosomes exhibited both GFP and RFP simultaneously, however LC3 in autolysosomes only showed red fluorescence due to the acidic environment in the lysosome lumen, which affected the GFP signal. The merged picture of Huh-7 cells infected with HBx lentivirus showed a clear and noticeable increase in red and yellow puncta. Nevertheless, the use of small interfering RNA (si-RRM2) to knock down RRM2 resulted in the disappearance of fluorescent puncta, particularly those that were yellow. This indicated that RRM2 knockdown hindered the flow of autophagy and primarily hindered the creation of autophagosomes (Fig. 7C, D). Similarly, LO2-HBx-siRRM2 cells and Huh-7-HBx-siRRM2 cells contained less GFP-LC3B puncta formation and lipidation than LO2-HBx cells and Huh-7-HBx cells, respectively (Fig. 7E, F). Ultrastructural analysis demonstrated typical autophagosomes in LO2-HBx cells and Huh-7-HBx cells, but autophagosomes were scarce after the transient transfection of si-RRM2 (Fig. 7G, H). All these results indicated that RRM2 blockade impaired the autophagy process induced by HBx.

### HBx promoted HCC cell tumorigenesis via RRM2

**Fig. 8:**
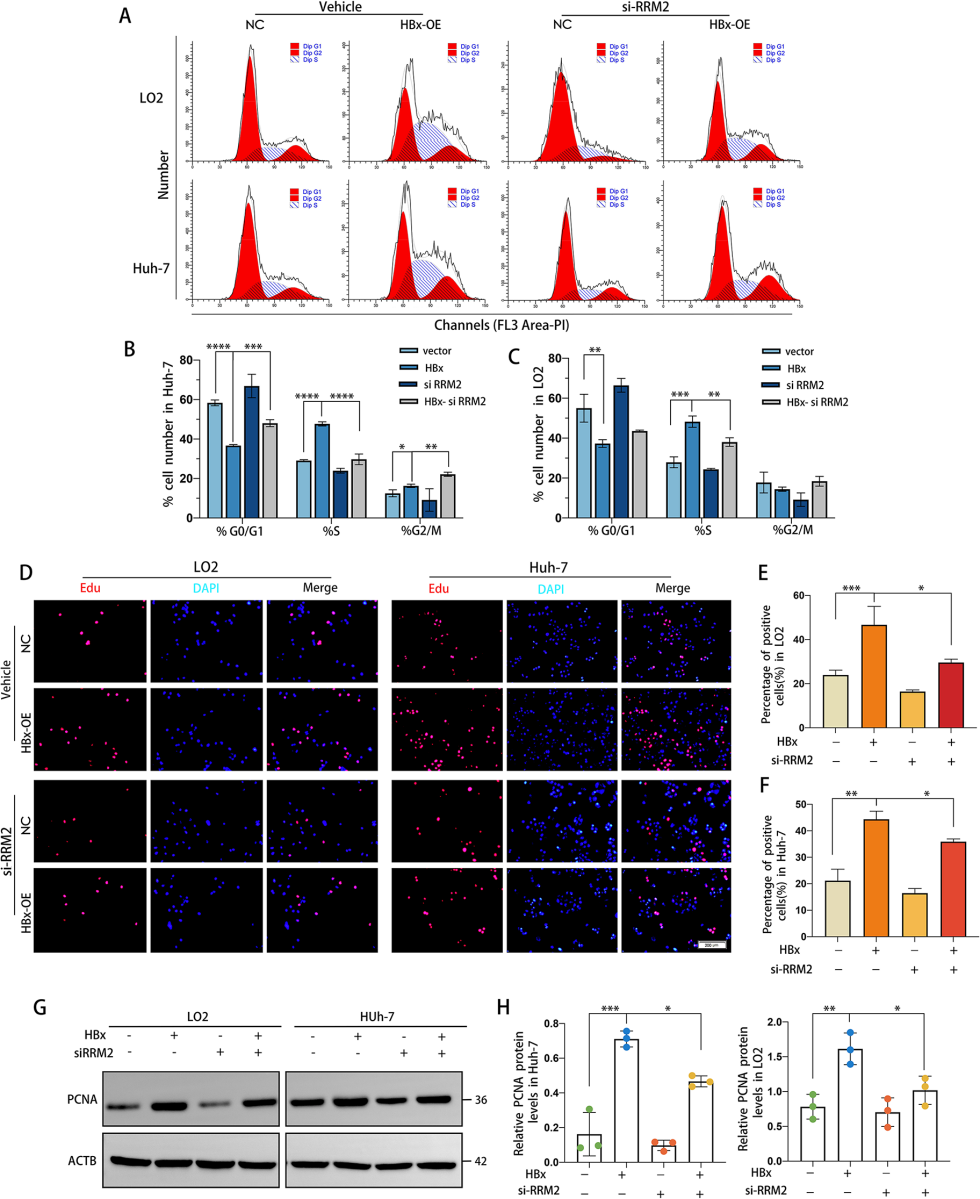
HBx promoted HCC cell proliferation via RRM2. LO2 or Huh-7 cells transfected with either LV-HBx or LV-NC plasmids were treated with/without siRRM2 for 48 h. (**A**-**C**) Analysis of cell cycle distribution among different groups by flow cytometry. **(D)** EdU incorporation assay was used to assess cellular proliferation following treatment. Scale bar: 200 μm. (**E**, **F**) Quantitative analysis of EdU-positive cells. **(G)** WB analysis of PCNA protein expression in LO2 or Huh-7 cells with the above treatments. **(H)** Quantitative of the relative PCNA protein expression in cells (*n* = 3). *P < 0.05, **P < 0.01, ***P < 0.001, ****P < 0.0001

**Fig. 9:**
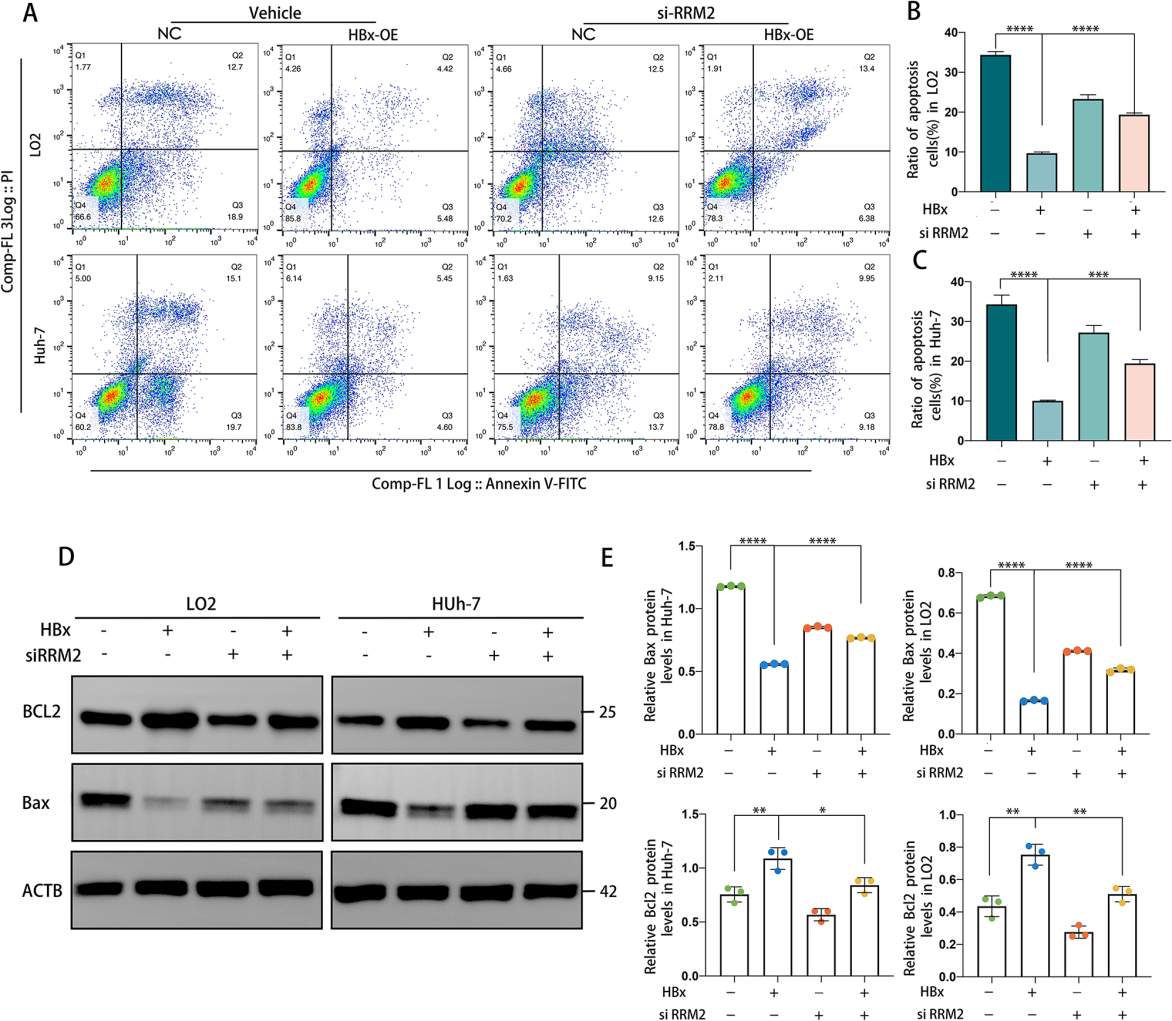
HBx promoted HCC cell apoptosis via RRM2. LO2 or Huh-7 cells transfected with the HA-HBx construct or empty NC were treated with/without siRRM2 for 48 h. **(A)** Analysis of apoptotic cells by flow cytometry. Representative Annexin V-FITC/PI-stained flow cytometry plots for apoptosis. (**B**, **C**) The proportion of apoptotic cells was displayed on the right panel. **(D)** WB analysis of BCL2 and Bax protein expression in LO2 or Huh-7 cells with the above treatments. **(E)** Quantitative of the relative BCL2 and Bax protein expression in cells (*n* = 3). *P < 0.05, **P < 0.01, ***P < 0.001, ****P < 0.0001

In many varieties of cancer, autophagy is inextricably linked to the cell cycle, whereas abnormal cell proliferation results from cell cycle changes. Since RRM2 is an intermediate mediator of HBx-induced autophagy, does RRM2 partake in HBx-regulated cell cycle regulation by affecting the cell cycle? For this reason, we utilized flow cytometry to examine the alterations in the cell cycle following RRM2 expression interference. Our data demonstrated that HBx-expressing cells rapidly exited the G0/G1 phase, entered the S phase, and nearly tripled their proliferation rate compared to HBx-deficient cells, whereas the si-RRM2 treatment inhibited the effect of HBx on proliferation (Fig. 8A-C). The results of EdU staining confirmed this conclusion. EdU experiments demonstrated that HBx overexpression boosted cell proliferation, but this effect was reversed after RRM2 interference (Fig. 8D-F). In addition, RRM2 knockdown downregulated PCNA, a factor related to DNA replication, indicating that HBx-induced stimulation of hepatocyte proliferation was dependent on RRM2 (Fig. 8G, H). Meanwhile, to determine whether RRM2 was required for HBx-induced inhibition of hepatocyte apoptosis, we examined the level of apoptosis in LO2-HBx and Huh-7-HBx cells after RRM2 knockdown using flow cytometry with Annexin V and PI staining. As depicted in Fig. 9A-C, more significant apoptosis was observed in LO2-HBx and Huh-7-HBx cells treated with si-RRM2. As depicted in Fig. 9D and E, overexpression of HBx enhanced the protein expression level of BCL2 and decreased the protein expression level of Bax, whereas treatment with si-RRM2 attenuated this effect. The aforementioned findings suggest that HBx promotes hepatocarcinogenesis via RRM2, with the mechanism involving the regulatory role of RRM2 in cell proliferation and apoptosis.

## Discussion

HCC is a highly widespread malignant neoplasm affecting the digestive system. It has been identified as the seventh most commonly occurring cancer globally and ranks as the third main cause of mortality associated with tumors. According to the Global Cancer Report, liver cancer is responsible for about 850,000 newly diagnosed cases and around 600,000 fatalities annually. Moreover, a significant majority, ranging from 70 to 80%, of these individuals are afflicted with liver cancer that is connected with the HBV. At now, surgical resection and liver transplantation are widely acknowledged as the fundamental therapeutic approaches for HCC. Nevertheless, it is important to acknowledge that the efficacy of surgical intervention is limited, with only a modest 15 to 25% of patients experiencing positive outcomes. Furthermore, a substantial 70% of patients who experience a recurrence following resection face an unfavorable prognosis, so significantly exacerbating the global healthcare burden [30, 31]. Cirrhosis typically represents the terminal phase of disease progression preceding the onset of HCC, and serves as one of the risk factors associated with the development of HCC. Furthermore, due to the absence of pain feeling in the liver, individuals with cirrhosis do not exhibit evident clinical symptoms along the progression from cirrhosis to liver cancer. This phenomenon results in a substantial number of people being identified with liver cancer during the intermediate or advanced stages of the disease, hence presenting a significant obstacle to the clinical management of liver cancer patients [32, 33].

Despite notable progress in the field of liver cancer treatment, the overall survival rate for those diagnosed with liver cancer continues to be disconcertingly low. The primary factor contributing to this scenario is the insufficient comprehension of the fundamental mechanisms involved in the genesis and progression of liver cancer. There exists a substantial body of evidence derived from epidemiological and functional investigations that supports a causal association between the abnormal expression of oncogenes and the onset of HCC. By conducting a comparative analysis of the disparities between tumorous and non-tumorous conditions, as well as examining variations in gene expression profiles across different phases of the disease, valuable research concepts and avenues for investigating the aberrant pathways and target molecules involved in HCC can be identified [34, 35].

Prior studies have established that HBV infection has a notable impact on the advancement of HCC in individuals with cirrhosis. HBV, functioning as an oncogenic virus, represents a significant risk factor in the pathogenesis of HCC. Infection with this virus elicits a chronic inflammatory response and excessive cell growth in the liver. This process is influenced not only by common risk factors like genetics, infection, and nutrition, but also by multiple pro-cancer pathways regulated by the HBV [36]. These factors collectively contribute to a detrimental cycle of liver inflammation, compensatory cell growth, and tissue damage, ultimately resulting in the advancement of the disease from hepatic fibrosis to cirrhosis and HCC [37–40]. Hence, it would be advantageous to explore the regulatory mechanisms behind the interaction between HBx and HCC, as well as the subsequent impact of HBx on downstream target molecules. This investigation has the potential to facilitate the identification of oncogenes that facilitate HBV-related HCC, as well as create a robust correlation between genes and the various stages of disease progression in relation to their oncogenic properties.

This study initially examined the expression profiles of RRM2 in HCC and HBV-related HCC using TCGA, GEO, and other databases. The aim was to investigate the expression pattern of RRM2 during the progression from cirrhosis to HCC and explore the relationship between its expression level in HCC and the pathological stage of the disease. The results of these analyses were then validated using molecular biology based on clinically relevant liver tissue samples. The findings from the data analysis revealed a frequent overexpression of RRM2 in primary HCC tissues, particularly in those associated with HBV. Moreover, the expression level of RRM2 was significantly higher in HBV-positive HCC tissues compared to HBV-negative HCC. These results suggest a potential association between the high expression of RRM2 in HCC and the presence of HBx. It is worth mentioning that RRM2 exhibited a discernible pattern of heightened expression levels during the progression from cirrhosis to HCC disease stage. This observation provides additional evidence that RRM2 plays a role not only in HBV-related HCC, but also that its elevated expression holds clinical significance in the assessment of HCC. Furthermore, our laboratory’s previous exploratory analyses have revealed the presence of RRM2 proteins in extracts of HBx-associated proteins derived from both LO2-HBx cells and Huh-7-HBx cells [17]. Based on the integration of these investigations, it is postulated that there exists a potential interaction between HBx and RRM2, which collectively contribute to the progression of hepatocarcinogenesis through the formation of protein complexes.

Kaplan-Meier analysis and Cox proportional risk model were employed to thoroughly evaluate the association between RRM2 expression and the survival duration of patients diagnosed with HCC. The examination of K-M survival curves revealed that RRM2 exhibits prognostic potential as a biomarker in individuals diagnosed with HCC. The findings from both univariate and multivariate Cox regression analyses indicate a potential correlation between elevated expression of RRM2 and decreased OS, DSS, and PFI in HCC. The examination of the ROC curve indicated that RRM2 exhibited superior diagnostic effectiveness. The aforesaid data imply that the high expression of RRM2 is closely associated to the development of HCC.

The accumulation of gene mutations contributes to the development of cancer to a certain degree. In light of this rationale, we conducted an investigation into the genetic alterations of RRM2 within tumor samples of HCC. Our study revealed that amplification is the predominant mutation type observed in HCC for the RRM2 gene. Subsequently, a pathway analysis was conducted on genes known to be associated with RRM2, revealing a notable enrichment of RRM2 and its associated genes across various carcinogenic pathways.

The uncontrolled growth of tumor tissues is not solely attributed to the intrinsic biological characteristics of tumor cells, but rather arises from the ongoing interplay between the cells within the organism and the microenvironment of the body. To this end, we constructed a hepatic in situ xenograft tumor animal model using Huh-7-HBx cells, a kind of HBx-overexpressing HCC cells, validated the stimulatory effect of HBx on HCC cell tumorigenesis in vivo, and analyzed the changes in the level of cellular autophagy during this process. We found that the degree of HCC was significantly increased in the HBx overexpression group of nude mice, and the degree of autophagy in liver tissue was significantly increased. Meanwhile, we investigated the relationship between “HBx and autophagy” and “autophagy and HBx-regulated cell proliferation and apoptosis” based on the HBx stable overexpression cell model. Our study revealed that the overexpression of HBx led to alterations in the cellular morphology, including the development of autophagic vesicles. Additionally, there were modifications in the expression of intracellular autophagy signature proteins, indicating an augmentation in the level of intracellular autophagy. In contrast, the administration of the autophagy inhibitor 3-MA resulted in the reversal of both the cell proliferation induced by HBx and the associated apoptotic effect. This finding effectively supports the conclusion that HBx can play a role in regulating tumor cell proliferation and apoptosis by stimulating autophagic fluxes, thereby promoting hepatocarcinogenesis.

Based on the aforementioned experimental findings, we proceeded to investigate the potential regulatory association between HBx and RRM2, as well as the biological significance of RRM2 in the route of HBx-induced hepatocarcinogenesis. Our findings indicate that the expression level of RRM2 was significantly elevated in HCC cells compared to normal hepatocytes. Additionally, there was a further increase in RRM2 expression in HBV-positive HCC cells compared to HBV-negative HCC cells. In a parallel manner, we noted a consistent pattern of RRM2 expression in an animal model featuring hepatic in situ xenograft tumor. The hepatic tissue of nude mice in the HBx-OE group exhibited a notably elevated expression level of RRM2 in comparison to the Vector control group. The aforementioned findings indicate that HBx exerts a regulatory influence on the expression of RRM2, aligning with the outcomes derived from our investigation conducted on clinical samples.

Si-RRM2 was employed to modulate the expression of RRM2, followed by an examination of the cellular processes of autophagy, proliferation, and apoptosis subsequent to the interference with RRM2 expression. To our surprise, the expression of fluorescent spots, autophagosomes, and the autophagy signature protein exhibited varying degrees of reduction and depletion following the inhibition of RRM2 expression in the cells. These findings suggest that the inhibition of RRM2 weakens the level of autophagy induced by HBx. In this study focused on HBV-realted HCC, our research establishes a novel association between autophagy and RRM2. Notably, we demonstrate for the first time that the regulation of autophagy by HBx is contingent upon the expression of RRM2. Furthermore, the analysis conducted by Edu and WB revealed that the cell proliferation phenomenon, facilitated by HBx, seemed to be inhibited upon intervention in RRM2 expression. This inhibition was observed not only in the decrease of cellular DNA replication activity but also in the expression of PCNA, a protein associated with proliferation, within the cells. These findings suggest that HBx indirectly stimulates cell proliferation by inducing elevated expression of RRM2.

Several autophagy-related genes have been shown to exert an influence on the cell cycle, which serves as the internal and meticulous governing mechanism for cell growth [35]. To elucidate the underlying mechanism by which RRM2 promotes abnormal proliferation of HCC cells, we conducted an investigation on the impact of inhibiting RRM2 expression on the cell cycle regulated by HBx. Flow cytometry was employed to analyze the data, revealing that the absence of RRM2 expression induced a G1/S-phase block, consequently reducing the rate of cell proliferation. The findings obtained from our experiment align with the proposition made by Lei Y et al. that disruption of autophagy leads to the emergence of G1/S blockage in cells, consequently leading to a reduction in the rate of cell proliferation induced by HBx [29]. In addition, Annexin V-FITC/PI double staining assay and WB analysis showed that inhibition of RRM2 expression also impaired, to some extent, the resistance of cells to death in the presence of HBx. The aforementioned findings indicate that RRM2 functions as an upstream regulator of autophagy in the HBx-mediated autophagy-promoted hepatocarcinogenesis oncogenic pathway. So, what mechanisms does HBx employ to enhance the expression level of RRM2? In order to achieve this objective, we initially employed indirect double immunofluorescence labeling to examine the spatial distribution of RRM2 in relation to HBx in HCC tissues associated with HBV. The findings demonstrated a partial co-localization between the two proteins. CO-IP analysis also showed that HBx was tightly linked to the RRM2 counterpart protein. To further investigate whether HBx causes high RRM2 expression from the transcriptional level, we performed CUT&TAG analysis, a research method to probe mRNA-protein interactions. Regrettably, the CUT&TAG analysis revealed that the chromatin associated with HBx does not exhibit direct binding to the mRNA of RRM2. This observation implies that the ability of HBx to modulate the expression of RRM2 is not achieved through direct binding to the promoter region of the RRM2 gene at the transcriptional level.

Collectively, our findings demonstrate a previously unidentified association between autophagy, RRM2, and the formation of HCC associated with HBV. Specifically, we have established that RRM2 plays a role in the process of carcinogenesis through its dependence on autophagy, whereas HBx enhances the expression of RRM2. Given the importance of RRM2 in HBV-related HCC, our findings provide new insights into the molecular regulatory mechanisms of autophagy and carcinogenesis in HBV-related HCC.

## Materials and methods

### Transient transfection, generation of stable cell lines and RNA interference

The Ubi-HBx-SV40-Neomycin plasmid, which carries inserts encoding HBx, was cloned into the vector CV084 which is a consequence of optimization derived from commercial vector pfu-GW (GeneChem, China). To generate stable HBx-overexpression cell lines (LO2-HBx, Huh-7-HBx), LV-HBx (GeneChem, China) were constructed and used for corresponding cells by lentivirus-mediated transfection [35, 39]. Parameters: MOI_huh−7_=5, MOI_LO2_=4. For two weeks prior to the ensuing studies, stable transfections were chosen and treated with G418 (GeneChem, China). Small interfering RNA siRRM2 (5ʹ-GGAGGAGAGAGUAAGAGAATT-3ʹ) targeting the coding region of the respective mRNA, were used according to the manufacturer’s instructions (Hanbio, China).

### Bioinformation analysis

The current exploration of RRM2 expression used the Cancer Genome Atlas database (TCGA, https://portal.gdc.com, accessed on 6th October 2022), a microarray cancer database with a web-based data mining platform, and the analysis data came from LIHC Level3 HTSEQ-FPKM RNA seq [41, 42]. The validation of RRM2 expression levels in HCC were verified using microarray data from GEO database (https://commonfund.nih.gov/GTEx/) (accessed on 4th October 2022). The data for the mRNA expression were taken from GSE10143 [38] (80 HCC tissues and 225 hepatitis cirrhosis tissues), GSE54236 (80 liver cirrhosis and HCC tissues from the same patients), GSE25097 (6 healthy liver tissues, 40 cirrhosis tissues, 222 HCC tissues and para-cancer tissues) [41] and GSE17548 (10 HBV-related HCC tissues and 13 HBV-related cirrhosis tissues) datasets [37]. To assess the level of RRM2 expression, the “R-limma” and “R-edgeR” packages were employed. The " R-ComplexHeatmap " [Version 3.3.3] package was used to visualize the expression profile. Additionally, we examined the methylation signature and the association between RRM2 and clinicopathological characteristics using the UALCAN database (https://ualcan.path.uab.edu/) [42]. To identify RRM2-related signaling pathways, GSEA was used to identify the gene sets that displayed statistically significant differences between high-RRM2 and low-RRM2 groups, using a normalized enrichment score (NES) of > 1.5 and a nominal *P* value of 0.05 as the threshold [43].

### Clinical samples and cell lines

From the parahemangioma sites of 8 patients with hepatic hemangiomas who were free of the hepatitis virus, normal liver tissue samples were taken. Ten cirrhotic samples were taken from HBV-infected cirrhotic patients undergoing liver transplantation. 151 paired HCC and paracancerous tissue specimens were obtained from HCC patients undergoing surgery, 85 of whom had HBV infection. Histology was then utilized to confirm all of the samples. For the 148 patients who were regularly observed, the median duration of follow-up since diagnosis was 47 months (range 1–139 months). All patients provided their informed consent and the Huashan Hospital Human Ethics Committee authorized the experimental protocols (IRB: 2021 − 783). The Chinese Academy of Sciences’ Institute of Biochemistry and Cell Biology (Shanghai, P.R. China) provided the immortalized normal liver cell line HL-7702 (LO2) and three human HCC cell lines (Huh-7, HepG2, HepG2.215). They were cultured in high-glucose Dulbecco’s modified Eagle’s medium (DMEM/F12, BI) with 10% fetal bovine serum (FBS) at 37 °C. The autophagy inhibitor 3-Methyladenine (3-MA, 5 mM) was obtained from MedchemExpress (MCE).

### Mice and treatments

Animal care, including all surgical procedures, was conducted in compliance with the ethical norms. Six-week-old male BALB/c nude mice from the Chinese Science Academy were used in the mouse orthotopic xenograft model. On the subepithelial surface of the diaphragm of the mouse liver’s left lobe, 5 × 10^6^/100 µL of cell solution comprising Huh-7 liver cancer cell lines transfected with either LV-HBx or LV-NC was slowly injected [44–46]. Day 0 marked the inoculation of tumor cells. Following the inoculation of tumor cells, routine monitoring included recording the effect of tumor growth on the usual behavior of the animals and the weight of the mice. Six weeks after injection, the mice were sacrificed, and the tumor nodules were harvested. All experimental procedures were authorized by the Committee on the Ethics of Binzhou Medical University (approval no.2020027).

### RT-PCR

TRIzol (invitorgen, USA) was utilized to isolate total RNA. Utilizing the reverse transcription kit (Takara, Japan), cDNA was generated. Real-time PCR was conducted using the SYBR Premix Ex TaqTMII kit (Takara, Japan). β-actin was used as an internal control.

Related primers sequence:

RRM2 forward -CATTGTGAGGTACAGGCGGAAG-3’.

reverse-GAAATGGTCTGAGCTGGCAGAAG-3’.

HBx forward -TACCGTCCCTTGCTTTCTCT-3’.

reverse-CAGAGGTGAAGCGAAGTGC-3’.

β-actin forward -TGGCACCCAGCACAATGAA-3’.

reverse- CTAAGTCATAGTCCGCCTAGAAGCA-3’.

### Western blotting

After lysing the total protein in each group of cells in RIPA lysis buffer including proteinase inhibitors, the protein concentrations in each group were measured using a BCA Protein Assay Kit (Keygentec, Nanjing, China). HBx (Genetex, 22741), RRM2 (ABclonal, A3424), SQSTM1/p62 (Proteintech, 18420), LC3B (Proteintech, 14600), Bax (ABclonal, A0207), BCL2 (ABclonal, A0208), PCNA (ABclonal, A0264), and ACTB were used for Western blotting (WB). PVDF membranes with proteins were incubated with primary antibodies at 4 °C overnight and corresponding secondary antibodies at room temperature for 2 h. The quantitative densitometry of the specified proteins was normalized to ACTB.

### Coimmunoprecipitation assay

Coimmunoprecipitation (Co-IP) assays were performed on Huh-7-HBx and LO2-HBx cells. These beads were washed three times with lysis buffer. As a control, the antibodies (Beyotime, A7016 and A7028 IgG) from the matching species were utilized. Following SDS-PAGE separation, the immunoprecipitations were analyzed by WB.

### Cell growth assay

5-ethynyl-2’-deoxyuridine (EdU) tests are utilized to measure cell proliferation. The cells were exposed to EdU (Ribobio, China) for two hours as instructed by the manufacturer. The cells were observed using a fluorescent microscope.

### Transmission electron microscopy

The cells were fixed, embedded in 2% agarose, and cut into 1 mm cubes before being fixed overnight. Then, the cells were fixed in 0.1 M cacodylate buffer containing 1% OsO4, dehydrated, and embedded in Spurr’s resin (Electron Microscopy Sciences, 14,300), which was subsequently sliced into 60-nm ultrathin sections and examined with a Hitachi-H7000 transmission electron microscope (S/N 747–32–03; Rapid City, SD, USA). Transmission electron microscopy was used to detect autophagic structures. The volume fraction of autophagic compartments in each section was quantified using random sampling [47].

### Flow cytometric analysis

The cell cycle was evaluated by staining the cells with propidium iodide (PI; Beyotime). FITC annexin V isothiocyanate (FITC) and PI were used to measure apoptosis by staining cells. Flow cytometry (FACScan; BD Biosciences) was used to assess harvested cells in accordance with the manufacturer’s instructions. Profiles of cell-cycle distribution and the proportion of apoptotic cells were evaluated using Modfit LT 3.2.

### Immunohistochemical and immunofluorescence analysis

Liver tissues were fixed immediately after isolation with 4% paraformaldehyde and then embedded in paraffin. On paraffin sections, immunohistochemistry and immunofluorescence staining were performed. For tissue staining, slides were treated with primary antibody against HBx (Genetex, 22741), RRM2 (ABclonal, A3424), or SQSTM1/p62 (Proteintech, 18420). The sections were counterstained with hematoxylin following immunohistochemical staining. Using the positive number of pixels and color deconvolution method of Image-Scope software, the puncta and percentage of positive area in 5 fields of each tissue sample were calculated. For immunofluorescence staining, cells were incubated with HBx (Genetex, 22741), RRM2 (Abcam, ab32099), or LC3B (Proteintech,14600) primary antibody and detected by corresponding secondary antibodies. DAPI (Invitrogen, D1306) was used to stain nuclei.

#### Tandem stubRFP-sensGFP-LC3 fluorescence microscopy

Tandem stubRFP-sensGFP-LC3 lentiviruses (GeneChem, China) were utilized to observe the flow of autophagy. To evaluate tandem fluorescent LC3 puncta, 48 h after transfection of tandem stubRFP-sensGFP-LC3 lentiviruses alone or in combination with si-RRM2 into Huh-7-HBx cells, the cells were washed three times with PBS and then fixed with 4% paraformaldehyde. The nuclei were stained with DAPI. The images were acquired using a confocal microscope.

### Statistical analysis

The results are expressed as mean ± standard deviation (SD) values. A statistical analysis was performed using GraphPad Prism 6.01 and R language 3.6.2 version. Statistical significance was calculated with Student’s *t*-test between two selected groups or one-way ANOVA analysis in comparisons of multiple groups. Using the Kaplan-Meier survival curve and the log-rank test, overall survival in relation to RRM2 expression in HCC patients was examined. By calculating the receiver operating characteristic (ROC) and the area under the curves (AUCs), the diagnostic value of RRM2 was obtained using the “time ROC” and “ggplot2” R packages [16]. We used the “R-forestplot” and “R-survival” packages to analyze and illustrate the univariate and multivariate Cox regression analysis. Using the “R-survival”, “R-rms”, and “R-ggplot2” packages, we developed a nomogram based on the best multivariate Cox regression analysis to forecast the probabilities of survival for 1-year, 3-year, and 5-year. Assessing the calibration plot using the “rms” software. To examine the usefulness of the model, the decision curve analysis (DCA) was evaluated using the “R-survival” package.

## Electronic supplementary material

Below is the link to the electronic supplementary material.

Supplementary Material 1

## Data Availability

All of the databases utilized in this study can be accessed through online public databases. The published article contains all data sets generated/analyzed for this research. All the data supporting the findings of this study are available within the paper. Data will be made available on reasonable request, not applicable for material.
