# Supplementary material for: HBx promotes tumorigenicity through RRM2-mediated autophagy in hepatocellular carcinoma
Source: Cell Biosci. 2024 Sep 10;14:116. doi: 10.1186/s13578-024-01298-2 (PMC11389268; doi:10.1186/s13578-024-01298-2)

Figure S1 GEO databases were used to validate the expression of RRM2 in liver cirrhosis and HCC tissues. HCC tissues group vs. cirrhosis tissues group in GSE25097 **(A),** HCC tissues group vs. cirrhosis tissues group in GSE10143 **(B),** HBV-related HCC tissues vs. HBV-related cirrhosis tissues group in GSE17548 **(C),** HCC tissues group vs. cirrhosis tissues group in GSE54236 **(D).** Green and red represents cirrhosis and HCC samples, respectively. GEO, Gene Expression Omnibus. ****P* < 0.001.


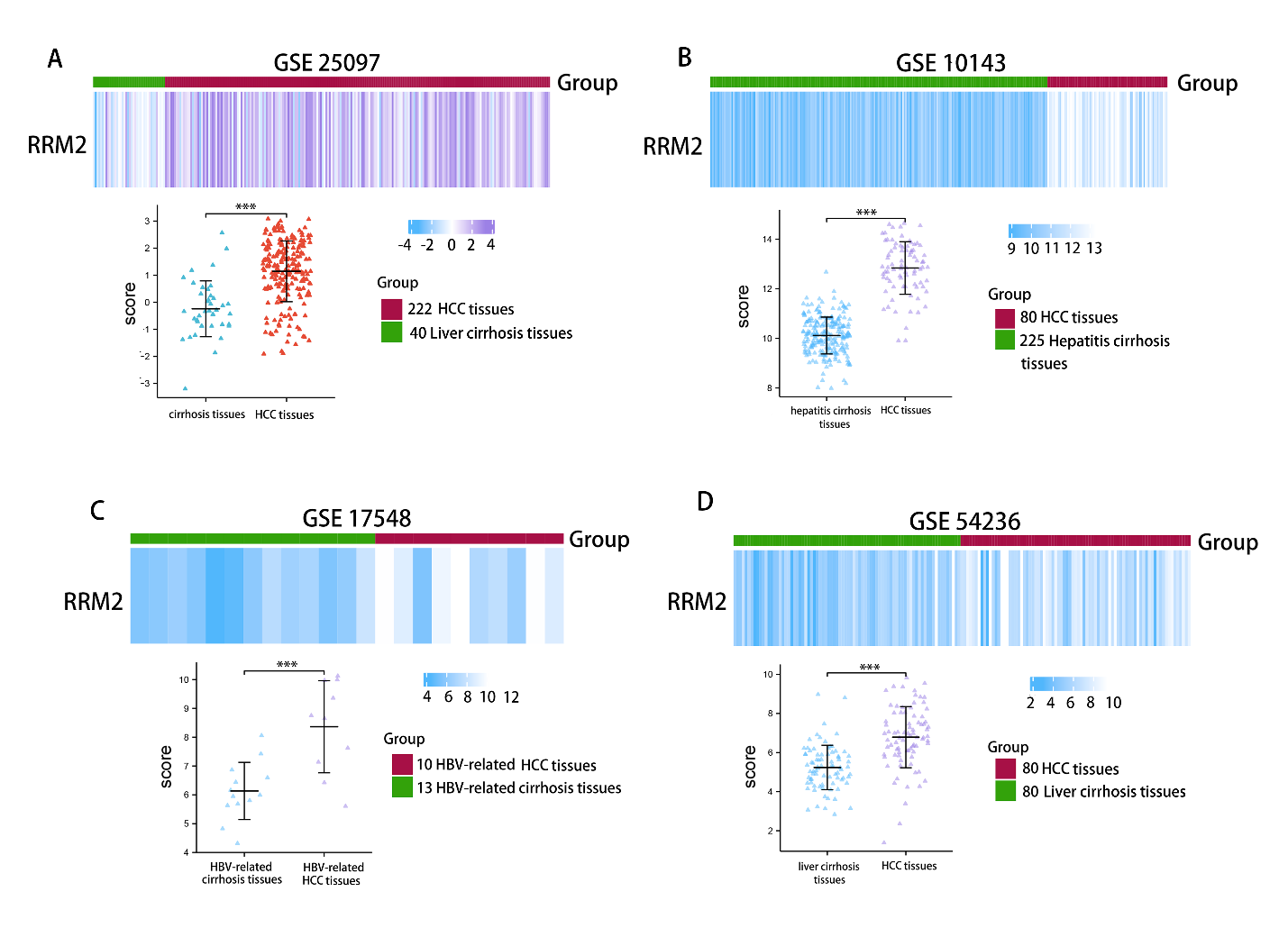


Figure S2 Alteration frequency of RRM2 in HCC from cBioPortal database.

(A) OncoPrint visual summary of alteration on a query of RRM2. (B) Summary of alteration in RRM2. (C) RRM2 expression in different RRM2 CNV groups. (D-G) Kaplan-Meier plots comparing RRM2 gene alterations with OS, PFS, and DSS of HCC patients.


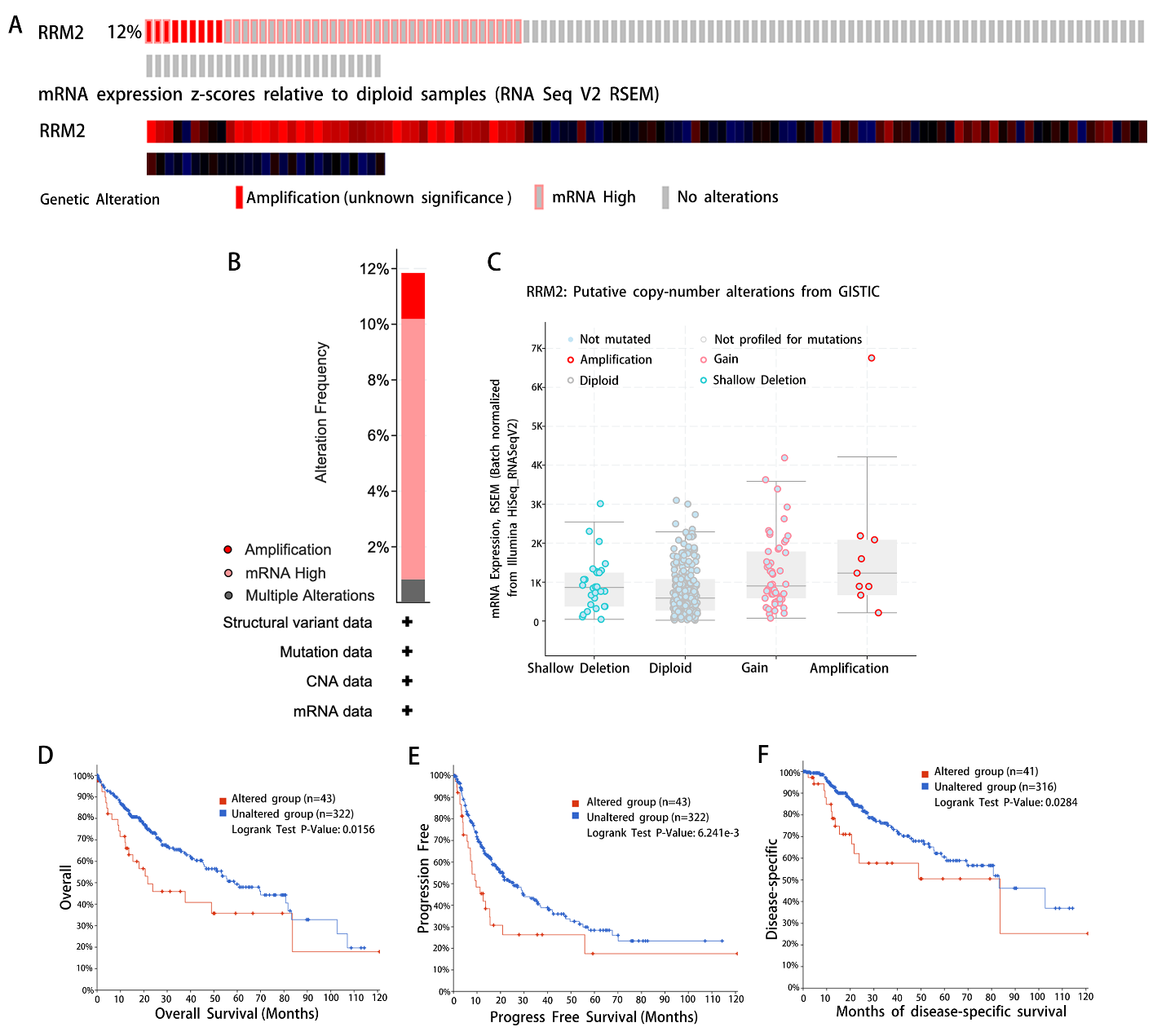

Supplement: Supplementary file 1 — Supplementary Material 1 [file 13578_2024_1298_MOESM1_ESM.docx]
